# Length of lags in responses of milk yield and somatic cell score on test day to heat stress in Holsteins

**DOI:** 10.1111/asj.13186

**Published:** 2019-02-27

**Authors:** Koichi Hagiya, Ikumi Bamba, Takefumi Osawa, Yamato Atagi, Naozumi Takusari, Fumiaki Itoh, Takeshi Yamazaki

**Affiliations:** ^1^ Department of Life and Food Science Obihiro University of Agriculture and Veterinary Medicine Obihiro Japan; ^2^ Data Analysis Division National Livestock Breeding Center Nishigo Fukushima Japan; ^3^ Department of Agricultural and Environmental Biology Graduate School of Agricultural and Life Sciences The University of Tokyo Tokyo Japan; ^4^ NARO Hokkaido Agricultural Research Center Sapporo Japan

**Keywords:** heat stress, Holstein, temperature–humidity index, test day

## Abstract

We used daily records from provincial Japanese weather stations and monthly test‐day records of milk production to investigate the length of the lags in the responses of cows’ milk yield and somatic cell score (SCS) to heat stress (HS). We also investigated the HS thresholds in milk yield and SCS. Data were a total of 17,245,709 test‐day records for milk and SCS in Holstein cows that had calved for the first time between 2000 and 2015, along with weather records from 60 weather stations. Temperature–humidity index (THI) values were estimated by using average daily temperature and average daily relative humidity. Adjusted THI values were calculated by using temperature, relative humidity, wind speed, and solar radiation. The model contained herd, calving year, month of test day, age group, days in milk, and THI as a fixed effect. THIs for each day from 14 days before the test day until the test day were used to represent the HS effects. The HS occurring 3 days, and between 8 and 10 days, before the test day had the greatest effect on the milk yield and SCS, respectively. The threshold THI values for the HS effect were about 60–65 for both traits.

## 1 INTRODUCTION

Negative effects of heat stress (HS) in dairy cows have been widely investigated because of economic losses in the dairy industry globally. The HS affects feed intake, yield, and reproduction (e.g., Hayes, Carrick, Bowman, & Goddard, 2003; West, Mullinix, & Bernard, 2003). Public weather stations supply useful information for studies of HS in dairy cattle (Ravagnolo, Misztal, & Hoogenboom, 2000). The temperature–humidity index (THI) is generally used to estimate the effects of HS. Test‐day milk yield decreases by about 0.2 kg per unit increase in THI (Ravagnolo et al., 2000). A high THI has been associated with increased somatic cell score (SCS) in several studies (Hammami, Bormann, M'hamdi, Montaldo, & Gengler, 2013; Lambertz, Sanker, & Gauly, 2014). Hammami et al. (2013) evaluated indices of the effects of HS on milk, fat, protein, and SCS; they reported the superiority of using a THI that was adjusted for wind speed and solar radiation, as reported by Mader, Davis, and Brown‐Brandl (2006).

Heat stress (HS) in Holstein cattle is an issue of growing concern for the dairy industry in Japan. Nagamine and Sasaki (2008) investigated the effects of temperature on fertility of Holstein–Friesian cattle in Japan. They found that temperature had highly significant negative effects on conception rates in southern Japan. Hagiya et al. (2017) reported that negative seasonal effects on SCS and conception ratio at first service were larger in southern Japan than in central and northern Japan. Atagi et al. (2017) reported seasonal changes in semen production traits with changes in temperature and humidity data from national weather stations.

In previous studies, a delayed response of test‐day yield to HS was reported. Hammami et al. (2015) assigned a THI averaged over the 3 days before the test day for yield traits and SCS in their analysis of HS effects in Luxembourg and Germany. Bernabucci et al. (2014) reported that the greatest negative effect on yield traits (milk, fat, and protein yields and fat and protein percentages) in Italian Holsteins was observed when the HS occurred 4 days before the test day. Carabaño et al. (2016) used the average THI of the test day and the two preceding days in their analysis of HS, to take into account the length of the lag in the response to HS in Holsteins in Belgium, Luxembourg, Slovenia, and Spain; the results varied across countries.

The number of studies of SCS response to HS is limited compared with those of milk yield and components. Moreover, to our knowledge, no published reports have used THI to investigate the length of the lag in the response of SCS to HS in Japan or northeast Asia.

Here, our main aim was to elucidate the lengths of the lags in responses of milk yield and SCS to HS in cows by using test‐day records and daily weather records from provincial weather stations in Japan. We also investigated the threshold values of THI in milk yield and SCS.

## 2 MATERIALS AND METHODS

### 2.1 Data

Test‐day records of milk and somatic cell count (SCC) at 6 through 305 days in milk in Holstein cows that had calved for the first time between 2000 and 2015 were provided by the Livestock Improvement Association of Japan (Tokyo, Japan). Records were collected through the Dairy Herd Improvement Program. The data included records of test‐day milk yield and SCC for first‐lactation cows from all over Japan. The SCCs were log‐transformed into SCSs by using the following formula (Ali & Shook, 1980):

$$\text{SCS}=\log_{2}\left(\mathrm{SCC}/100000\right)+3.$$

Weather records from 60 provincial weather stations for the period from 2000 to 2015 were obtained from the website of MeteoCrop DB (an agro‐meteorological database coupled with crop models; Institute for Agro‐Environmental Science, 2017). Ravagnolo et al. (2000) reported that maximum daily temperature and minimum daily relative humidity were the most critical variables in calculating the THI to quantify the HS. However, in our preliminary study using weather records in Japan, we found that the THI based on average temperature and average relative humidity in a day was more effective than that calculated by using maximum temperature and lowest relative humidity in a day. Therefore, THI values were estimated by using average daily temperature and average daily relative humidity. First, the THI was estimated by using the following formula (NRC, 1971):

(1) THI=1.8×t+32(0.55−0.0055×rh)×(1.8×t−26),

where *t* is the temperature in degrees Celsius and *rh* is the relative humidity as a percentage. In the preliminary study, we estimated the HS effect as the THI adjusted for a daily average wind speed and solar radiation in a day (Mader et al., 2006). However, the AICs with adjusted THI were similar to those with THI. That is, no superiority of adjusted THI over THI was observed under Japanese weather conditions.

A total of 17,245,709 test‐day records from 2,018,406 cows were used. Five subsets (divided randomly by herd because of computing memory limitations) were analyzed separately in the cases of milk yield and SCS. Mean daily milk yield was 27.1 kg and mean SCS was 2.33 (Table 1). Mean THI ranged from 50.7 to 51.0 (Table 2). The minimum THI was 4; the respective maximum value was 84.

**Table 1:** Means, standard deviations (*SD*), and minimum and maximum values for milk yield and somatic cell score (SCS)

Trait/subset	*N*	Mean	*SD*	Minimum	Maximum
Milk yield (kg/day)					
1	3,526,655	27.1	6.5	0.2	98.0
2	3,221,502	27.0	6.6	0.2	75.9
3	3,450,506	26.9	6.3	0.2	74.7
4	3,532,269	27.2	6.4	0.2	81.8
5	3,514,777	27.2	6.4	0.2	79.0
Somatic cell score					
1	3,526,655	2.33	1.65	−3.64	11.17
2	3,221,502	2.34	1.64	−3.64	11.33
3	3,450,506	2.33	1.64	−3.64	11.90
4	3,532,269	2.33	1.65	−3.64	12.52
5	3,514,777	2.32	1.64	−3.64	11.29

**Table 2:** Means, standard deviations (*SD*), and minimum and maximum values for temperature–humidity index (THI) and THI adjusted for wind speed and solar radiation (THI_adj_)

Trait/subset	*N*	Mean	*SD*	Minimum	Maximum
THI					
1	3,526,655	51.0	15.1	4	84
2	3,221,502	50.7	15.0	4	84
3	3,450,506	50.9	15.0	4	84
4	3,532,269	51.0	15.1	4	84
5	3,514,777	51.0	15.2	4	84

### 2.2 Model

Test‐day records were linked to the data from provincial weather stations in the 14 branches in Hokkaido, which is a northern island in Japan, and in the other 46 prefectures. The effects of HS were estimated by using a statistical model, as follows:

(2) $$y_{\mathrm{ijklmn}}=H_{i}+Y_{j}+M_{k}+A_{l}+{\text{DIM}}_{m}+{\text{THI}}_{n}+e_{\mathrm{ijklmn}}$$

where *y*_*ijklmn*_ is an observation of test‐day milk or SCS; *H*_*i*_ is the fixed effect of herd *i*;* Y*_*j*_ is the fixed effect of year at calving *j* (16 subclasses); *M*_*k*_ is the fixed effect of month *k* (12 calendar months); *A*_*l*_ is the fixed effect of age group *l* (18–20, 21 and 22–35 months); DIM_*m*_ denotes the days in milk *m* (300 subclasses); THI_*n*_ is the index of HS as expressed by THI *n* (81 subclasses); and *e*_*ijklmn*_ represents vectors of random residual effects. THIs for any single day from 14 days before the test day until the test day were used to represent the HS effects. When a model did not contain the fixed effects of HS, it was assumed to be a basic model. Akaike's information criterion (AIC) and the least‐squares mean (LSM) within each of five subsets were estimated by using the GENMOD procedure for AIC, or the generalized linear model (GLM) procedure for the LSM (SAS Institute, 2016) and compared among models with different THIs. The fitness of each subset was compared with the difference of the AIC for the basic model. Analyses were conducted separately for each of the five subsets, and means and standard errors were calculated from these results.

**Figure 1:**
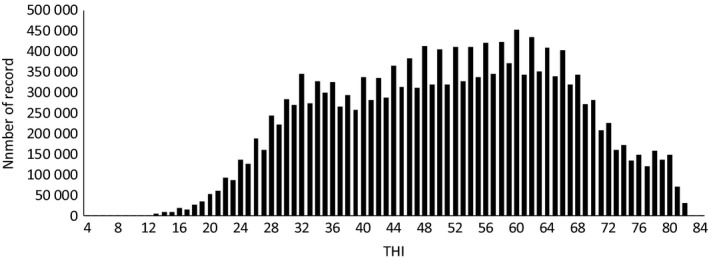
The number of records in the temperature–humidity index (THI)

Assuming that the effect of HS was linear, the breakpoint of THI was estimated by segmented‐regression analysis by using the Segmented package (Muggeo, 2003, 2008) of (R Core Team, 2014). In a way similar to the method of Carabaño et al. (2016), the LSMs of THI effects in Equation (2) were used as the dependent variables and the slope of the segmented linear regression at lower than the breakpoint was assumed to be 0, as follows:

$$y_{i}^{*}=c+e_{i};\;\;\text{when}\;\;x_{i}\leq \text{BP},\;\;\text{and}$$

$$y_{i}^{*}=a+b*x_{i}+e_{i};\;\;\mathrm{when}\;\;x_{i}>\text{BP},$$

where $y_{i}^{*}$ is the LSM of the THI effect estimated by using 3 (or 8) days before test day in milk yield (or SCS), c is a constant, *a* is an intercept, *b* is a regression coefficient on THI *x*_*i*_, and *e*_*i*_ is the random residual term. BP is the breakpoint, defined as the appropriate threshold value of THI when the linear regression was applied to the HS effect. The number of records on THI is shown in Figure 1. We used THI classes with more than 20,000 records for segmented‐regression analyses owing to the stability of LSM estimates (THI, ranging from 18 to 82).

## 3 RESULTS

### 3.1 Length of lags in response to HS

**Figure 2:**
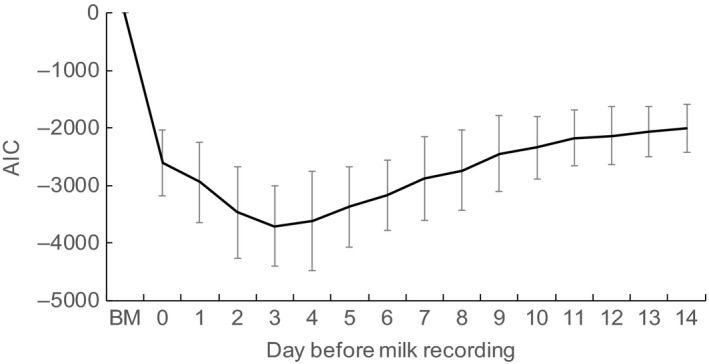
Estimates of Akaike's information criterion (AIC) and 95% confidence intervals (bars) for milk yield, as affected by the temperature–humidity index during the days before the test day. Basic Model without the effects of heat stress (HS)

**Figure 3:**
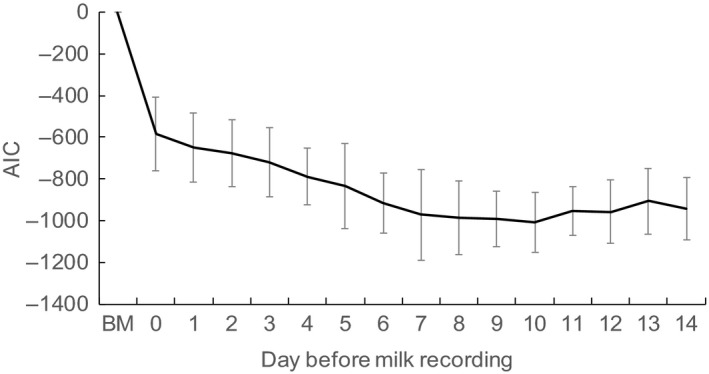
Estimates of Akaike's information criterion (AIC) and 95% confidence intervals (bars) for somatic cell score (SCS), as affected by the temperature–humidity index during the days before the test day. Basic Model, basic model without the effects of heat stress

When we used THI to model the effect of HS, the estimated AIC for milk yield decreased sharply from test day to 3 days before the test day and then increased toward 14 days before the test day (Figure 2). For SCS, the estimated AIC with HS using THI decreased gradually from the test day to 7–10 days before the test day and then increased a little toward 14 days before the test day (Figure 3).

### 3.2 Least‐squares means for the effect of heat stress

**Figure 4:**
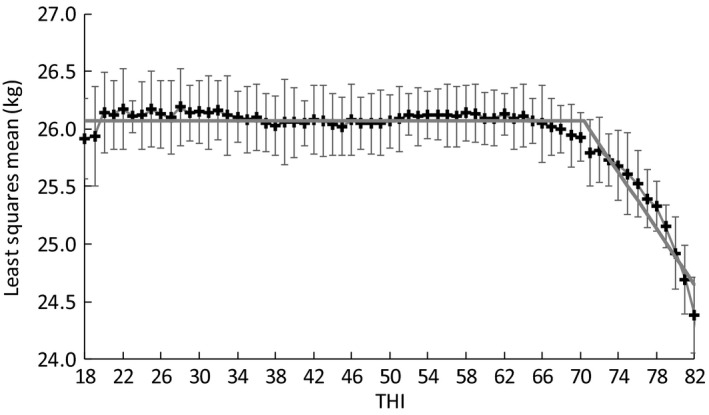
Least‐squares means (LSM) and 95% confidence intervals (bars) for the effects of heat stress (HS) (as indicated by changes in the temperature–humidity index during the 3 days before the test day, THI) on milk yield, and lines estimated by segmented‐regression analysis

**Figure 5:**
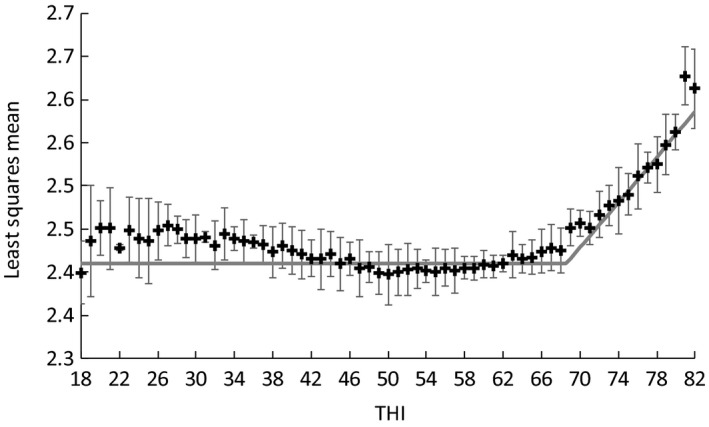
Least‐squares means (LSM) and 95% confidence intervals (bars) for the effects of heat stress (HS) (as indicated by changes in the temperature–humidity index of the 8 days before the test day, THI) on somatic cell score (SCS), and lines estimated by segmented‐regression analysis

When the effect of HS was assumed to be linear, the estimates of THI breakpoint were 70.4 for milk yield and 68.5 for SCS. When the values of THI exceeded a threshold, the LSM for the effects of HS on milk yield in the 3 days before the test day decreased gradually with increasing THI (Figure 4). The graphed changes in the effects of HS on milk yield were quadratic, and the threshold values for the THI ranged from about 60 to 65 THI for milk yield. For SCS, the LSM increased with increasing THI beyond a threshold (Figure 5); the threshold THI values were again in the range from about 60 to 65. The LSMs were increased moderately when the THI values were less than 45.

## 4 DISCUSSION

Daily milk yield and SCS were in agreement with those recently reported (26.9 kg for daily milk yield and ranging from 2.3 to 2.5 for SCS) in Holstein cows in Japan (Hagiya et al., 2017; Yamazaki et al., 2016).

Bohmanova, Misztal, Tsuruta, Norman, and Lawlor (2008) found in their preliminary study that the weather data 3 days before the test day explained more of the variability in milk yield than the data on the 2 days before the test day or on the test day itself. Bernabucci et al. (2014) reported that the greatest negative effect was observed when the HS occurred 4 days before the test day. West et al. (2003) similarly reported that milk yield was affected by the THI as recorded 2 days before the test day. Hayes et al. (2003), in contrast, found in preliminary investigations of their data that the THI on the test day and 1, 2, 3, and 4 days before the test day had significant effects on test‐day yield. Here, we found no significant differences between the estimated AIC for milk yield using the THI 3 days before the test day and those from 2 to 6 days before the test day. Our finding here of a critical effect of the THI 3 days before the test day on milk yield was thus generally in line with these previous results.

In their analysis of HS effects, Hammami et al. (2015) and Santana, Bignardi, Pereira, Stefani, and El Faro (2016) used the THI averaged over the 3 days before the test day to examine the effects on yield traits and SCS. Smith, Smith, Rude, and Ward (2013) also used the averaged THI for the 3 days before the test day to estimate the effect of HS on SCS in Holstein and Jersey cows. Here, we found no significant differences between the AIC estimated for SCS by using the THI 10 days before the test day and those from 5 to 14 days before the test day. However, our results showed a longer lag time for SCS than milk yield, with the greatest response to HS between 7 and 10 days before the test day. Dry matter intake (DMI) is sensitive to the mean air temperature 2 days earlier than the test day (West et al., 2003), and it causes a decrease in the availability of nutrients used for milk synthesis (Polsky & von Keyserlingk, 2017). Maintenance energy requirements are increased during heat stress because of the high energy levels required for heat loss from the body during heat stress (Atrian & Shahryar, 2012). These effects lead to a negative energy balance, resulting in a decrease in milk.

A negative effect of HS on yield traits is found when the THI reaches 72 (Ravagnolo et al., 2000). The HS thresholds for a THI over 69–72 have been reported for various milk traits (Carabaño, Ramon, Diaz, Lolina, & Perez‐Guzman Serradilla, 2017). Hammami et al. (2013) reported that the THI threshold value was 62 for milk yield. By using the method of Hayes, Bowman, Chamberlain, Verbyla, and Goddard (2009), Nguyen, Bowman, Haile‐Mariam, Nieuwhof, and Hayes (2017) estimated a genetic evaluation of heat tolerance with a threshold of THI 60. Bernabucci et al. (2014) reported that the threshold varied among studies; they concluded that this variation may have occurred because of variations in the methods used to detect the THI thresholds. They also suggested that threshold values were affected by the type of herd cooling system, such as fans or sprinklers. Carabaño et al. (2017) concluded that estimates of thresholds can vary with climatic regions and milk production levels. In recent reports, relatively low THI thresholds, ranging from 60 to 65, have been reported for HS effects on yield traits (e.g., Ammer, Lambertz, & Gauly, 2016; Nguyen, Bowman, Haile‐Mariam, Pryce, & Hayes, 2016). Our THI thresholds seemed to be about 60 and 65 for both traits—similar to the values in these recent reports. However, further studies on HS thresholds are needed to elucidate quadratic changes in the effect of HS on milk yield. For SCS, when the THI values were less than 45, the estimated effects of THI appeared higher than when the THI values were around 50 (see Figure 5); the optimal THI for Holstein cows seemed to be around 50. However, the differences between the effects of low THIs and moderate THIs were quite small. For SCS, Bertocchi et al. (2014) reported that the slope (0.0003 × THI) on THI was small before the breakpoint. Our results were in line with their trends, and the breakpoints we obtained seemed to be somewhat higher than the THI thresholds. However, the breakpoints estimated here may be applicable when the genetic merit of animals for heat tolerance is estimated by using a random regression model (Atagi et al., 2018; Ravagnolo & Misztal, 2000; Santana et al., 2016).

We investigated the lengths of the lags in responses of milk yield and SCS to HS in cows by using test‐day records and daily records from provincial weather stations in Japan. The HS occurring 3 days before the test day had the greatest effect on milk yield. For the effect on SCS, there was a longer lag in the response to HS: the peak response was when the HS occurred between 7 and 10 days before the test day. The threshold THI effect values of thresholds were about 60–65 for both traits.

## ACKNOWLEDGMENT

This work was supported by a grant from the Ministry of Agriculture, Forestry and Fisheries of Japan (Development of Breeding Technology for Animal Life Production).
